# Effect of Shenfu Injection on Porcine Renal Function after Cardiopulmonary Resuscitation

**DOI:** 10.1155/2020/3789268

**Published:** 2020-04-22

**Authors:** Shen Zhao, Ziren Tang, Hao Cui, Zhengfei Yang, Fei Shao, Shuo Wang, Feng Chen

**Affiliations:** ^1^Shengli Clinical Medical College, Fujian Medical University, Fuzhou 350001, China; ^2^Department of Emergency Medicine, Fujian Provincial Hospital, Fujian Institute of Emergency Research, Fuzhou 350001, China; ^3^Department of Emergency Medicine, Beijing Chao-Yang Hospital, Capital Medical University, Beijing 100020, China; ^4^Beijing Key Laboratory of Cardiopulmonary Cerebral Resuscitation, Beijing Chao-Yang Hospital, Capital Medical University, Beijing 100020, China; ^5^Department of Emergency Medicine, Sun Yat-sen Memorial Hospital, Sun Yat-sen University, Guangzhou, China

## Abstract

**Objective:**

To comprehensively evaluate the protective effect of Shenfu injection (SFI) on renal ischaemia/reperfusion injury (IRI) after cardiopulmonary resuscitation (CPR) through neutrophil gelatinase-associated lipocalin (NGAL) and to explore effective monitoring of early renal injuries after CPR.

**Methods:**

Thirty healthy minipigs were randomly divided into 3 groups: sham operation (SO) (*n* = 6), control (*n* = 12), and SFI (*n* = 12). The SO group underwent only catheterization, whereas the control and SFI groups were subjected to program-controlled electrical stimulation to establish a cardiac arrest (CA) model due to ventricular fibrillation. After CPR, the return of spontaneous circulation was achieved. Each animal in the SFI group was intravenously injected with SFI after resuscitation. Haemodynamic parameters were monitored at baseline and 2, 6, 12, and 24 hr after CPR. At each time point, venous blood samples were collected for NGAL, creatinine, and ATPase screening.

**Results:**

After CA, the MAP, CPP, and CO of the animals in the control and SFI groups decreased significantly. However, at 6 hr after CPR, the MAP, CPP, and CO of the animals in the SFI group began to recover gradually; the differences between the control and SFI groups were significant (*P* < 0.005). The renal damage immediately after CPR appeared to be significant in the pathological examinations. However, the degree of renal injury in the SFI group improved significantly, and the apoptosis index was also notably reduced. The blood and urine NGAL levels were clearly elevated after CPR. The greatest increase in NGAL was found in the control group, which was significantly different from that of the SFI group (*P* < 0.001). SFI can significantly increase the ATPase activity of kidney tissues after CPR and improve abnormal caspase-3 protein expression.

**Conclusion:**

SFI can effectively prevent acute kidney injuries caused by CPR through improving energy metabolism and inhibiting apoptosis.

## 1. Introduction

With continuous improvement of living standards and ageing of the population, the incidence of cardiovascular and cerebrovascular diseases has been increasing annually. The most common cause of death due to cardiovascular incidents is cardiac arrest (CA). In the United States, the estimated number of patients suffering from CA outside the hospital is 300,000 per year. Even with successful cardiopulmonary resuscitation (CPR), the survival rate of a patient after discharge remains very low, with a median of 4.1% [1]. Deaths after CPR usually are due to failure of important organs caused by systemic ischaemia/reperfusion injury (IRI), such as the heart, brain, or other vital organs [2], among which kidney failure is among the common outcomes.

Shenfu injection (SFI) is a traditional Chinese medicine solution that is prepared through modern medical processing techniques based on the traditional recipe of a Chinese medicine called “Shenfu soup.” SFI is often used to treat shock syndromes, heart failure, and arrhythmia. Animal studies have provided evidence that SFI can increase the CPR success rate [3–5]. In this study, we used the quick, highly sensitive, and specific diagnostic marker neutrophil gelatinase-associated lipocalin (NGAL) to detect acute kidney injury and performed a comprehensive analysis of the protective effect of SFI on renal IRI after CPR. The study aimed to provide a theoretical basis and experimental data for discovery of more efficient measures to monitor early CPR-related renal injuries and improve renal functions.

## 2. Materials and Methods

### 2.1. Animal Preparation

Thirty healthy male Wuzhishan (WZS) minipigs were purchased from the Beijing Laboratory Animal Research Centre (license number: SCXK 11-00-002). The minipigs were aged from 12 to 14 months with body weights of approximately (30 ± 2) kg. One day before surgery, the minipigs underwent fasting with free access to water. After anaesthesia was induced by injecting 2 mg·kg^−1^ propofol in the ear vein, the animal was bound on the operating table in a fixed supine position. The first dose of the anaesthetic drug (3% pentobarbital sodium) was intravenously injected at 30 mg·kg^−1^·h^−1^ and then maintained at 8 mg·kg^−1^·h^−1^. A 6.5 F tracheal intubation tube was placed and connected to a ventilator (Drager-Evata IV, Dräger, Germany) to assist breathing. The ventilator parameters were set as follows: inhaled oxygen concentration, 21%; ventilation frequency, 12 times/min; and initial tidal volume (TV), 15 mL/kg. The end-tidal carbon dioxide partial pressure (ETPCO2) and TV were monitored using a respiratory monitor (CO2SMOplus, Respironics, USA). The respiratory rate and TV were adjusted to maintain the ETPCO2 within the range of 35 to 40 mmHg. After skin preparation, the animal was connected to the electrocardiogram (EKG) machine (HP-M1165, Hewlett Packard, USA) for electrocardiography. A 7 F sheath was placed into the left internal jugular vein to facilitate placement of a temporary pacemaker with double electrodes all the way to the right ventricle. A 5 F arterial catheter was placed in the left femoral artery all the way to the ascending aorta, and the pressure transducer and HP monitor were connected to measure the aortic pressure (AOP). A 7 F floating catheter was placed in the left femoral vein all the way to the pulmonary artery to measure the right atrial pressure (RAP) and cardiac output (CO) and to collect blood samples.

### 2.2. Establishment of the Ventricular Fibrillation Model and Experimental Procedures

The animals were allowed to recover for 45 minutes after the operation and were divided into the following three groups using the random number table: sham operation (SO) (*n* = 6), control (*n* = 12), and SFI (*n* = 12). Pacemaker leads were inserted into the internal jugular vein catheters of the animals except for those in the SO group and connected to an external medical program-controlled stimulator (GY-600A, China Kaifeng Huanan Instrument Co., Ltd.) for CA. Ventricular fibrillation (V-fib) was induced in the animals by continuous electrical stimulating pulses at 10 ms in an 8 : 1 ratio [6]. The criteria for V-fib are a sudden rapid drop in arterial blood pressure and the appearance of V-fib waveforms on the electrocardiogram (ECG). After 8 minutes of V-fib, the standard CPR procedures began (i.e., 30 : 2 compression/aeration ratio at a frequency of 100 beats/min with a depth of 50 mm; the chest was allowed to fully rebound after relaxing, paused for 10 seconds after every 30 presses, and ventilated twice with an air artificial balloon at a TV of 300 mL each time). The CPR quality was monitored with the Q-CPR technology (HeartStart MRx Monitoring/Defibrillator, Philips, the Netherlands) [7]. After 2 minutes of CPR, the first defibrillation was performed with a two-way wave of 150 J (HeartStart MRx Monitoring/Defibrillator, Philips, the Netherlands). If unsuccessful, epinephrine (0.02 mg/kg) was intravenously administered, followed by another 2 minutes of CPR. The heart rhythm was monitored for 10 seconds between every 2 minutes of CPR. If restoration of spontaneous circulation (ROSC) did not occur after 4 defibrillation cycles, the CPR was stopped, and the animal was allowed to die. The criteria for ROSC are as follows: blood pressure recovers to normal, and the AOP exceeds 50 mmHg or the pulse pressure reaches >20 mmHg for a duration of at least 1 minute [8]. For the SFI group, 1 mL/kg of SFI was administered intravenously immediately after successful resuscitation of the animals; for the control group, normal saline solution was injected with the same volume. All animals received mechanical ventilation of 100% pure oxygen and were monitored for 4 hours. If hypotension or ventricular arrhythmia emerged, dopamine or amiodarone was administered. After 4 hours of monitoring, all tracheal intubations and other arteriovenous catheters were removed from the animals except for one set that was kept for venous access. Then, the minipigs were placed in cages and observed for 24 hours. All experimental procedures were the same among the groups except that the animals in the SO group did not receive V-fib inductions and CPR. All surviving animals were sacrificed after 24 hours of cage observation by intravenous injection of a combinational solution of overdosed anaesthesia drug and potassium chloride. Pathological specimens of the corresponding organs were collected immediately.

### 2.3. Monitoring the Haemodynamic Parameters

The heart rate (HR), mean arterial pressure (MAP), and CO at baseline and 2, 6, 12, and 24 hr after CPR were monitored and recorded. The CO was examined by injecting a salt solution (4°C) into the right atrium through a central venous catheter using the pulmonary thermodilution method [6]. Blood and urine samples were collected at the same time points for the creatinine and NGAL assays, and the urine volumes were recorded.

### 2.4. NGAL and Creatinine Levels in the Blood and Urine

The NGAL levels in the blood/serum and urine were measured using an enzyme-linked immunosorbent assay kit (Beijing New Equation Biotechnology Co., Ltd.) following the manufacturer's instructions. Each sample was tested twice, and the average was used as the final reading. The creatinine levels in the serum and urine were detected using a colorimetric assay (LabAssay™; Wako Pure Industrial Ltd., Japan) following the kit's instructions. The creatinine level in the samples was detected by the bright yellow colour generated through its reaction with picric acid in an alkaline solution (Jaffe reaction). The reaction's absorbance was measured by using a spectrophotometer at the 520 nm wavelength, and the creatinine concentration in the specimen was determined from the standard curve.

### 2.5. Measurement of Na^+^-K^+^-ATPase and Ca^2+^-ATPase Activity

After the animals were sacrificed, the kidney was dissected, and the Na^+^-K^+^ adenosine triphosphate enzyme (ATPase) and Ca^2+^-ATPase activities were measured using specific spectrophotometric kits (Nanjing Jiancheng Bioengineering Institute). One unit (U) of ATPase activity was defined as the amount of ATPase in each milligram of tissue protein that could decompose ATP to produce 1 *μ*mol of inorganic phosphorous per hour.

### 2.6. Western Blot Analysis

Western blotting was performed to examine the expression levels of related proteins (Bcl-2, Bax, and activated caspase-3). The myocardial tissues were collected from the left ventricular anterior wall, and the Bcl-2, Bax, and caspase-3 protein expression levels were evaluated by western blotting. The antibody concentrations used for the corresponding proteins, and their sources were as follows: Bax, 1 : 500 (Santa Cruz Biotechnology, USA); Bcl-2, 1 : 200 (EMD Millipore, USA); activated caspase-3, 1 : 500 (Abcam Biotechnology, UK); and GAPDH, 1 : 250 (Santa Cruz Biotechnology, USA).

### 2.7. Histological Testing and Grading of the Specimens

The tissue from the left upper pole of the kidney was removed and placed in 10% neutral formaldehyde solution. After paraffin-embedded fixation, dewaxing, sectioning, and haematoxylin and eosin (HE) staining, the pathological changes in the tissues were observed under a light microscope. Based on the previously reported method, semiquantitative evaluation of pathological changes was carried out under a microscope by professional pathologists [9]. The samples were graded according to the following standards: ***Grade A***: ≤25% of the renal tubular epithelial cells display degeneration or necrosis, with no detectable abnormalities in the glomeruli, stroma, and blood vessels but some chronic degeneration in ≤5 of the glomeruli and interstitium (fibrosis or sclerosis); ***Grade B***: >25% but ≤50% of the renal tubular epithelial cells display degeneration and necrosis, and/or ≤5% show glomerular autolysis and slight degeneration of the ball and interstitium (fibrosis or sclerosis) that is >5% but ≤10%; and ***Grade C***: ≥50% of the renal tubular epithelial cells display degeneration and necrosis together with glomerular and vascular endothelial cell degeneration and nuclear lysis, ≥10% show tubular basement membrane collapse and/or fracture, and ≥20% exhibit simple degenerative changes. Paraffin specimens from the left ventricle of the animals from each group were tested with the regular TDT-mediated biotinylated dUTP nick end-labelling (TUNEL) method following the instructions provided in the kit (Roche Diagnostics, Inc., Germany). The number of apoptotic cells and the total number of cells were counted five times under 200x magnification using a light microscope. The apoptosis index (AI) was calculated using the following formula: AI (%) = number of apoptotic cells/total cell number × 100%.

### 2.8. Statistical Analysis

The statistical analyses were performed using SPSS 10.0 software, and the data are expressed as the (mean ± standard deviation). The measurement data were analysed by analysis of variance of repeated measures, and the count data were analysed with the *χ*^2^ test. *P* < 0.05 was considered statistically significant.

## 3. Results

### 3.1. Evaluation of the Resuscitation Outcomes

All animals in the SO group survived the entire experimental procedure. One animal in the control group and one in the SFI group did not recover from the resuscitations. The active CPR period was (4.9 ± 1.2) min for the control group and (4.8 ± 1.3) min for the SFI group, which were not significantly different (*P* > 0.05).

### 3.2. Haemodynamic Parameters (Table 1)

No significant differences in the haemodynamic parameters were noted among the groups at baseline (*P* > 0.05). All parameters of the SO group animals remained stable throughout the experiment. The HR of the animals in the control and SFI groups experiencing CA increased significantly after resuscitation, whereas their MAP, CPP, and CO were reduced significantly. Six hr after CPR, the MAP, CPP, and CO of the SFI group animals began to recover gradually, and their differences from those of the control group animals became statistically significant (*P* < 0.005).

### 3.3. Renal Histology

**Figure 1:**
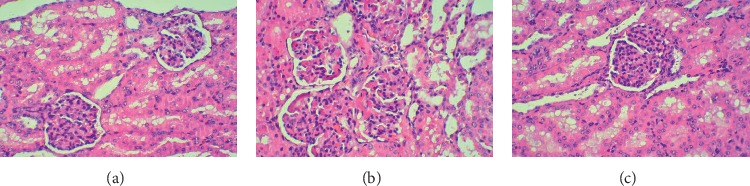
Haematoxylin and eosin (H&E) staining. The animals underwent CPR displayed signs of kidney damage with HE staining, including congestion in the renal tubular and glomerular capillaries, mesangial hyperplasia, inflammatory cell infiltration, and microthrombus formation in the renal arterioles (b) and (c). The renal injury in the SFI group was significantly reduced compared to that in the control group (c), whereas no obvious damage was found in the kidney pathology of the animals in the SO group (a). (a) The Sham operation group. (b) The control group. (c) The SFI group.

Under a light microscope, tissues from the animals that underwent CPR displayed signs of kidney damage, including congestion in the renal tubular and glomerular capillaries, mesangial hyperplasia, inflammatory cell infiltration, and microthrombus formation in the renal arterioles. The renal injury in the SFI group was significantly reduced compared to that in the control group, whereas no obvious damage was found in the kidney pathology of the animals in the SO group (Figure 1). The kidney damage in the SO group was classified as Grade A in all animals. The numbers of animals in the control group with different grades of renal injury were as follows: Grade A, 0; Grade B, 3; and Grade C, 9. For the SFI group, the grade classifications were as follows: Grade A, 1; Grade B, 9; and Grade C, 2. Compared with those of the control group, the number of animals with Grade C kidney damage in the SFI group was significantly decreased (*χ*^2^ = 7.425, *P*=0.012).

**Figure 2:**
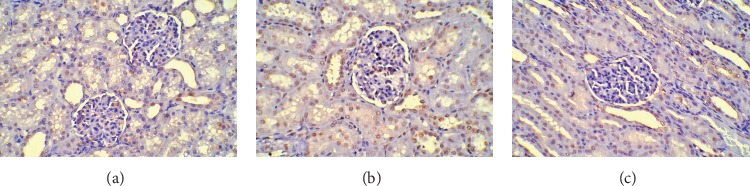
TDT-mediated biotinylated dUTP nick end-labelling (TUNEL). The apoptotic cells were stained with brown. Compared with that of the SO group, the AI of the control and SFI groups increased significantly (*P* < 0.01). Furthermore, when we compared the AI between the control and SFI groups, the AI of the SFI group was significantly reduced (*P* < 0.01). (a) The Sham operation group. (b) The control group. (c) The SFI group.

The AI for the SO, control, and SFI groups was (11.01 ± 0.3)%, (47.46 ± 18.67)%, and (27.37 ± 16.43)%, respectively. Compared with that of the SO group, the AI of the control and SFI groups increased significantly (*P* < 0.01). Furthermore, when we compared the AI between the control and SFI groups, the AI of the SFI group was significantly reduced (*P* < 0.01) (Figure 2).

### 3.4. Changes in Parameters Related to Renal Function

All parameters related to renal function remained stable in the SO group animals throughout the experiment. After resuscitation, the urine volumes of the control and SFI groups were significantly reduced compared with those of the SO group (*P* < 0.001). However, the urine volume of the SFI group gradually increased over time and became significantly different from that of the control group at 6 hours after CPR (*P* < 0.001). At 24 hr after CPR, the difference in the urine volume between the SO and SFI groups disappeared (*P* > 0.05). Slight increases in Cr after CPR were detected in each group, but the differences were not significant (*P* > 0.05). The blood and urine NGAL levels in the SFI and control groups were significantly increased after CPR compared to those of the SO group (*P* < 0.001). However, the increase in the NGAL level in the control group was more significant and was significantly higher than that of the SFI group (*P* < 0.001) (Table 2).

### 3.5. ATPase Activity

The Na^+^-K^+^-ATPase activity of the sham, control, and SF groups was (8.79 ± 1.07), (2.17 ± 1.45), and (5.46 ± 1.23) U/L, respectively; the Ca^2+^-ATPase activity of the three groups was (8.66 ± 1.31), (3.15 ± 1.24), and (5.678 ± 1.77) U/L, respectively. For both types of ATPase activity after CPR, a significant decrease was found in the control group (*P* < 0.01), but a significant increase was found in the SF group compared with that of the control group (*P* < 0.05).

### 3.6. Expression Levels of Proteins Related to Cardiomyocyte Apoptosis

**Figure 3:**
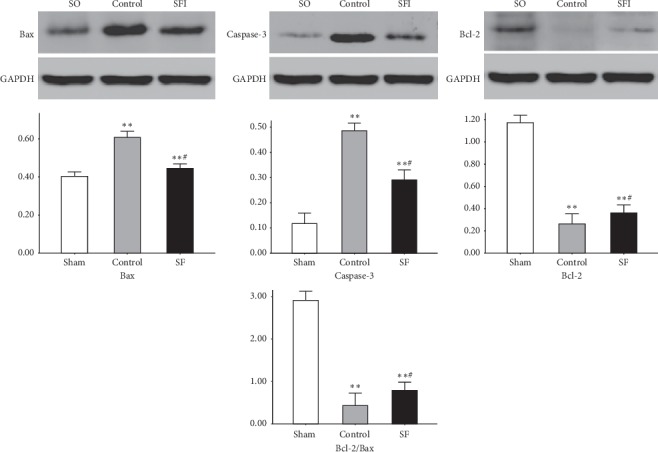
Western Blotting. The Bax and activated caspase-3 expression levels in the kidneys were significantly higher than those in the SO group. The Bcl-2 and Bcl-2/Bax levels were both significantly decreased (*P* < 0.01). The Bax and activated caspase-3 expression levels in the SFI group were also clearly reduced compared to those in the control group, whereas the Bcl-2 and Bcl-2/Bax expression levels were significantly increased by SFI (*P* < 0.05). ^*∗∗*^Versus the SO group, *P* < 0.01; ^#^vs. the control group, *P* < 0.05.

The Bax and activated caspase-3 expression levels in the kidneys were significantly higher in the SFI and control groups than in the SO group. The Bcl-2 and Bcl-2/Bax levels were both significantly decreased (*P* < 0.01). The Bax and activated caspase-3 expression levels in the SFI group were also clearly reduced compared to those in the control group, whereas the Bcl-2 and Bcl-2/Bax expression levels were significantly increased by SFI (*P* < 0.05) (Figure 3).

## 4. Discussion

The body responds to CA by redistributing the blood supply to ensure that enough blood flows to vital organs, such as the heart and brain. Reflexive contractions of the renal arteries accompanied by simultaneous ischaemia and hypoxia can lead to increased secretion of antidiuretic hormone and catecholamine in the body, which in turn cause decreased renal blood perfusion and reductions in the glomerular filtration rate (GFR) and urine volume, eventually resulting in glomerular and tubular damage. After the ROSC, the body's normal blood flow is restored, and the blood resumes supplying oxygen to tissues and organs throughout the whole body. The renal artery reflexively dilates as a response to the resumed blood supply, which can cause blood reperfusion injury in the kidney. Thus, regardless of the cause of CA, the kidneys can suffer damage caused by ischaemia/reperfusion reactions.

Cr and blood urea nitrogen (BUN) are traditional biomarkers used for the diagnosis and evaluation of renal functions. However, Cr is a less ideal marker in terms of sensitivity and promptness in detecting acute kidney injury. In fact, Cr only displays abnormalities after the kidney has lost more than 50% of its function. Thus, the above diagnostic methods are limited for the treatment of renal diseases. In recent studies, NGAL was identified as a quick biomarker for diagnosis of acute kidney injury (AKI) with excellent sensitivity and high specificity. When combined with other biomarkers, such as Cr, NGAL can significantly increase the diagnostic ability for AKI [10]. In the rat renal ischaemia-reperfusion model, urinary NGAL production was significantly upregulated within 2 hours of renal ischaemia [11]. The time window of the NGAL increase occurs 24–48 hours earlier than that of blood Cr after renal injury; thus, NGAL is a highly sensitive biomarker for early renal tubular injury that can be utilized to diagnose acute tubular damage from a variety of causes, such as nephrotoxic drugs, contrast agents, environmental toxins, and ischaemia [12]. The majority of CA-related kidney injuries, especially those caused by hypoxia, are renal tubular injuries. Therefore, detection assays combining NGAL, Cr, and BUN can reflect changes in renal functions quickly and more accurately and increase their diagnostic ability for kidney damage. In the animals used in our study, the NGAL levels in the blood and urine increased within 2 hours after CPR, whereas Cr did not show any significant changes until the end of the experiment. Therefore, NGAL is a more sensitive and valuable marker for assessing renal damage after CPR.

“Shenfu Soup” was originally documented in a medical book called “Jisheng Regimens” from the Song dynasty. It is mainly composed of ginseng and aconite and has been used for more than 800 years in China. SFI is an injectable solution of “Shenfu soup” that is prepared using modern extraction techniques. SFI has been applied for the clinical treatments of a variety of diseases, and its cardiovascular protective effects, such as dilating the coronary arteries, stabilizing blood pressure, reducing IRI, and improving cardiac function, are particularly impressive. At the same time, it can also enhance the body's immune functions [3–5]. The main components of SFI that have been identified by modern medical research are ginsenosides and aconitine alkaloids. Red ginseng and aconite have been proven to have multiple pharmacological effects, such as preventing ischaemia and hypoxia, scavenging oxygen-free radicals, inhibiting lipid peroxidation, reducing intracellular Ca^2+^ overload, and stabilizing membrane structures. Other benefits of SFI include lowering blood viscosity, improving the microcirculation, and reducing organ ischaemic injuries [13]. Our previous studies confirmed that SFI also had excellent effects on reducing peripheral resistance, pulmonary artery resistance, and pulmonary wedge pressure; increasing CO; and improving tissue perfusion and oxygen metabolism [14]. In this study, animals receiving SFI showed an increase in CO after CPR, which alleviated the haemodynamic effect of SVR. The increase in MAP was mainly due to the increase in CO. As the body's haemodynamics improved, renal perfusion was in turn alleviated, which was manifested as an increase in the urine volume and a decrease in the blood and urine NGAL levels. Other studies have suggested that some pharmacological effects of SFI, such as reducing organ dysfunctions and IRI and improving the body's haemodynamics after resuscitation, are related to its abilities to increase Na^+^-K^+^-ATPase and Ca^2+^-ATPase activities [15, 16]. The mechanisms associated with post-CPR renal injuries may be related to factors that occur after CPR, including disrupted energy metabolism, oxygen-free radical damage, intracellular calcium overload, and inflammatory mediator damage [17–19]. Consistent with the above findings, we noted that the Na^+^-K^+^-ATPase and Ca^2+^-ATPase activities decreased after CPR but increased after SFI treatment. The improvement due to SFI was manifested as reduced cell apoptosis in terms of the pathological changes in the organs, and its mechanism could be related to improvement of the aberrant expression levels of caspase-3 pathway proteins that regulate apoptosis.

## 5. Limitations

SFI solution is a composite blend of traditional Chinese medicines with a quite complicated formulation. The potential interactions between the various ingredients in the solution were not examined in this study. Furthermore, the results were only tested in animal models, and the effect of SFI in humans still needs to be confirmed in large-scale clinical trials.

## 6. Conclusions

In this study, we established a porcine CA model and applied SFI as a treatment intervention for renal injury after successful resuscitation. The highly sensitive, fast, efficient, and greatly specific NGAL assay was used to diagnose acute renal injury. After examining the structures, perfusion, function, metabolism, and pathological changes in the kidneys during the renal injury process after CPR, we were able to confirm the protective effect of SFI on CPR-related renal function damages. The mechanism may be associated with the ability of SFI to improve renal perfusion, increase ATPase activity, and regulate the caspase-3 pathway, thus ultimately reducing cellular apoptosis.

## Figures and Tables

**Table 1:** Haemodynamic parameters.

Groups	Baseline state	2 hr	6 hr	12 hr	24 hr
HR, times/min					
SO	110 ± 12	121 ± 18	112 ± 15	111 ± 15	112 ± 15
Control	107 ± 13	135 ± 22^*∗*^	132 ± 16^*∗*^	131 ± 17^*∗*^	138 ± 16^*∗∗*^
SFI	109 ± 15	136 ± 19^*∗*^	134 ± 14^*∗*^	132 ± 19^*∗*^	135 ± 11^*∗*^

MAP, mmHg					
SO	96 ± 6	99 ± 8	94 ± 9	94 ± 10	95 ± 9
Control	95 ± 5	75 ± 15^*∗∗*^	77 ± 17^*∗∗*^	75 ± 16^*∗∗*^	72 ± 15^*∗*^
SFI	94 ± 7	79 ± 13^*∗∗*^	86 ± 17^#^	87 ± 15^#^	86 ± 14^#^

CPP					
SO	90 ± 9	93 ± 11	91 ± 10	92 ± 11	90 ± 12
Control	89 ± 8	66 ± 15^*∗∗*^	67 ± 16^*∗∗*^	67 ± 14^*∗∗*^	66 ± 15^*∗∗*^
SFI	88 ± 8	70 ± 13^*∗∗*^	80 ± 11^*∗∗*^#	81 ± 12^*∗*##^	80 ± 10^*∗∗*^^##^

CO, L/min					
SO	3.3 ± 0.3	3.5 ± 0.4	3.2 ± 0.2	3.4 ± 0.3	3.1 ± 0.4
Control	3.1 ± 0.5	2.3 ± 0.9^*∗∗*^	2.2 ± 0.8^*∗∗*^	2.3 ± 0.7^*∗∗*^	2.3 ± 0.6^*∗∗*^
SFI	3.2 ± 0.4	2.4 ± 0.4^*∗∗*^	2.7 ± 0.5^*∗*#^	2.9 ± 0.4^*∗*#^	3.0 ± 0.5^#^

Systemic vascular resistance (SVR)					
SO	1050 ± 114	1201 ± 143	1198 ± 132	1212 ± 144	1184 ± 153
Control	1102 ± 121	1984 ± 225^*∗∗*^	2126 ± 276^*∗∗*^	2381 ± 366^*∗∗*^	2471 ± 343^*∗∗*^
SFI	1121 ± 131	1795 ± 302^*∗∗*^	2035 ± 249^*∗∗*^	2015 ± 328^*∗∗*^^#^	2004 ± 336^*∗∗*^^#^

^*∗*^ *P* < 0.05 compared with the SO group; ^*∗∗*^*P* < 0.01 compared with the SO group; ^#^*P* < 0.05 compared with the control group; ^##^*P* < 0.01 compared with the control group.

**Table 2:** Changes in renal function.

Groups	Baseline state	2 hr	6 hr	12 hr	24 hr
Urine volume (mL/kg/hr)					
SO	1.01 ± 0.12	1.00 ± 0.13	1.12 ± 0.12	1.14 ± 0.11	1.13 ± 0.13
Control	1.02 ± 0.11	0.41 ± 0.17^*∗∗*^	0.42 ± 0.19^*∗∗*^	0.39 ± 0.20^*∗∗*^	0.36 ± 0.21^*∗∗*^
SFI	0.99 ± 0.13	0.42 ± 0.15^*∗∗*^	0.87 ± 0.17^*∗∗*^^##^	0.89 ± 0.16^*∗∗*^^##^	0.93 ± 0.16^*∗*##^

Serum creatinine (sCr, *µ*mol/L)					
SO	62.12 ± 6.04	63.12 ± 5.36	61.26 ± 6.22	62.74 ± 5.68	63.54 ± 6.05
Control	63.24 ± 5.39	62.66 ± 6.98	66.45 ± 6.13	67.39 ± 7.12	67.12 ± 6.98
SFI	65.19 ± 5.39	61.34 ± 5.89	63.16 ± 6.61	66.13 ± 5.29	66.45 ± 6.01

Serum NGAL (sNGAL, ng/mL)					
SO	2.18 ± 0.12	2.21 ± 0.15	2.20 ± 0.13	2.19 ± 0.15	2.21 ± 0.16
Control	2.21 ± 0.11	4.11 ± 0.20^*∗∗*^	6.66 ± 0.19^*∗∗*^	8.19 ± 0.20^*∗∗*^	10.11 ± 0.15^*∗∗*^
SFI	2.19 ± 0.12	3.12 ± 0.21^*∗∗*^##	4.19 ± 0.18^*∗∗*^##	4.36 ± 0.21^*∗∗*^##	4.79 ± 0.22^*∗∗*^##

Urine NGAL (uNGAL, ng/mL)					
SO	0.49 ± 0.04	0.51 ± 0.07	0.50 ± 0.06	0.49 ± 0.05	0.52 ± 0.06
Control	0.50 ± 0.05	1.82 ± 0.09^*∗∗*^	4.45 ± 0.10^*∗∗*^	5.64 ± 0.12^*∗∗*^	6.87 ± 0.11^*∗∗*^
SFI	0.47 ± 0.06	1.43 ± 0.08^*∗∗*^##	1.99 ± 0.10^*∗∗*^##	2.01 ± 0.11^*∗∗*^##	2.14 ± 0.12^*∗∗*^##

^*∗*^ *P* < 0.05 compared with the SO group; ^*∗∗*^*P* < 0.01 compared with the SO group; ^#^*P* < 0.05 compared with the control group; ^##^*P* < 0.05 compared with the control group.

## Data Availability

The data used to support the findings of this study are available from the corresponding author upon request.

## References

[1] Navab E., Esmaeili M., Poorkhorshidi N. (2019). Predictors of out of hospital cardiac arrest outcomes in pre-hospital settings; a retrospective cross-sectional study. *Archives of Academic Emergency Medicine*.

[2] Neumar R. W., Nolan J. P., Adrie C. (2008). Post-cardiac arrest syndrome. *Circulation*.

[3] Zhang Q., Li C., Shao F., Zhao L., Wang M., Fang Y. (2017). Efficacy and safety of combination therapy of Shenfu injection and postresuscitation bundle in patients with return of spontaneous circulation after in-hospital cardiac arrest. *Critical Care Medicine*.

[4] Luo J., Min S., Wei K., Cao J. (2008). Ion channel mechanism and ingredient bases of Shenfu Decoction’s cardiac electrophysiological effects. *Journal of Ethnopharmacology*.

[5] Yuan W., Wu J.-Y., Wang G.-X., Zhang Q., Li C.-S. (2015). Effect of Shen-Fu Injection pretreatment to myocardial metabolism during untreated ventricular fibrillation in a porcine model. *Chinese Medical Journal*.

[6] Hamer A. W., Karagueuzian H. S., Sugi K., Zaher C. A., Mandel W. J., Peter T. (1984). Factors related to the induction of ventricular fibrillation in the normal canine heart by programmed electrical stimulation. *Journal of the American College of Cardiology*.

[7] Wu J.-Y., Li C.-S., Liu Z.-X., Wu C.-J., Zhang G.-C. (2009). A comparison of 2 types of chest compressions in a porcine model of cardiac arrest. *The American Journal of Emergency Medicine*.

[8] Yannopoulos D., Matsuura T., McKnite S. (2010). No assisted ventilation cardiopulmonary resuscitation and 24-hour neurological outcomes in a porcine model of cardiac arrest. *Critical Care Medicine*.

[9] Ma J., Li G., Xu W. (2013). The significance of zero-point biopsy in semi-quantitative scoring of frozen sections for the evaluation of DCD in renal injuries. *Chinese Journal of Organ Transplantation*.

[10] Han W. K., Wagener G., Zhu Y., Wang S., Lee H. T. (2009). Urinary biomarkers in the early detection of acute kidney injury after cardiac surgery. *Clinical Journal of the American Society of Nephrology*.

[11] Mishra J., Mori K., Ma Q., Kelly C., Barasch J., Devarajan P. (2004). Neutrophil gelatinase-associated lipocalin: a novel early urinary biomarker for cisplatin nephrotoxicity. *American Journal of Nephrology*.

[12] Westhuyzen J., Endre Z. H., Reece G. (2003). Measurement of tubular enzymuria facilitates early detection of acute renal impairment in the intensive care unit. *Nephrology Dialysis Transplantation*.

[13] Tang Z., Li C. S. (2005). Effect of Shen-fu injection on hemodynamics and oxygen delivery during hypovolemic shock. *Chinese Journal of Pathophysiology*.

[14] Yin W. P., Li C. S. (2008). Effect of Shenfu injection on hemodynamics and oxygen delivery metabolism in dogs with cardiogenic shock. *Chinese Journal of Integrated Traditional and Western Medicine in Intensive and Critical Care*.

[15] Ji X.-F., Yang L., Zhang M.-Y., Li C.-S., Wang S., Cong L.-H. (2011). Shen-Fu injection attenuates postresuscitation myocardial dysfunction in a porcine model of cardiac arrest. *Shock*.

[16] Lu Y., Wang S., Li C. S. (2012). Hypothermia ameliorates gastrointestinal ischemic injury sustained in a porcine cardiac arrest model. *Chinese Medical Journal*.

[17] Di Paola R., Genovese T., Impellizzeri D., Ahmad A., Cuzzocrea S., Esposito E. (2013). The renal injury and inflammation caused by ischemia-reperfusion are reduced by genetic inhibition of TNF-αR1: a comparison with infliximab treatment. *European Journal of Pharmacology*.

[18] Chalmers N. E., Yonchek J., Steklac K. E. (2020). Calcium/calmodulin-dependent kinase (CaMKII) inhibition protects against purkinje cell damage following CA/CPR in mice. *Molecular Neurobiology*.

[19] Madjene L. C., Danelli L., Dahdah A. (2020). Mast cell chymase protects against acute ischemic kidney injury by limiting neutrophil hyperactivation and recruitment. *Kidney International*.

